# Effects of Semantic Context and Fundamental Frequency Contours on Mandarin Speech Recognition by Second Language Learners

**DOI:** 10.3389/fpsyg.2016.00908

**Published:** 2016-06-14

**Authors:** Linjun Zhang, Yu Li, Han Wu, Xin Li, Hua Shu, Yang Zhang, Ping Li

**Affiliations:** ^1^Faculty of Linguistic Sciences and KIT-BLCU MEG Laboratory for Brain Science, Beijing Language and Culture UniversityBeijing, China; ^2^Department of Cognitive Science and ARC Centre of Excellence in Cognition and its Disorders, Macquarie UniversitySydney, NSW, Australia; ^3^Department of Sociology, Tsinghua UniversityBeijing, China; ^4^National Key Laboratory of Cognitive Neuroscience and Learning and IDG/McGovern Institute for Brain Research, Beijing Normal UniversityBeijing, China; ^5^Department of Speech-Language-Hearing Sciences and Center for Neurobehavioral Development, University of MinnesotaMinneapolis, MN, USA; ^6^Department of Psychology and Center for Brain, Behavior and Cognition, Pennsylvania State UniversityState College, PA, USA

**Keywords:** semantic context, fundamental frequency contours, speech recognition, second language (L2) proficiency, interfering speech

## Abstract

Speech recognition by second language (L2) learners in optimal and suboptimal conditions has been examined extensively with English as the target language in most previous studies. This study extended existing experimental protocols (Wang et al., 2013) to investigate Mandarin speech recognition by Japanese learners of Mandarin at two different levels (elementary vs. intermediate) of proficiency. The overall results showed that in addition to L2 proficiency, semantic context, F0 contours, and listening condition all affected the recognition performance on the Mandarin sentences. However, the effects of semantic context and F0 contours on L2 speech recognition diverged to some extent. Specifically, there was significant modulation effect of listening condition on semantic context, indicating that L2 learners made use of semantic context less efficiently in the interfering background than in quiet. In contrast, no significant modulation effect of listening condition on F0 contours was found. Furthermore, there was significant interaction between semantic context and F0 contours, indicating that semantic context becomes more important for L2 speech recognition when F0 information is degraded. None of these effects were found to be modulated by L2 proficiency. The discrepancy in the effects of semantic context and F0 contours on L2 speech recognition in the interfering background might be related to differences in processing capacities required by the two types of information in adverse listening conditions.

## Introduction

Speech recognition differences between native and non-native listeners have been examined extensively in previous studies, which have consistently showed that adults learning a second language (L2) are at a disadvantage in speech recognition as compared with native listeners, especially in adverse listening conditions (Gat and Keith, 1978; Mayo et al., 1997; Bradlow and Bent, 2002; Bradlow and Alexander, 2007; Golestani et al., 2009; Oliver et al., 2012). One class of explanations locates the primary source of the sharp decline in non-native listeners’ speech recognition in interfering backgrounds at the segmental level. On this account, native listeners have developed the ability to attend to segmental cues that are less vulnerable to interferer-related distortions, such as attending to formant transition cues to identify plosives when stop-burst information is masked by interfering sounds (Parikh and Loizou, 2005; Jiang et al., 2006). In contrast, non-native listeners may not develop such processing flexibility and have to attend primarily to cues that, while relatively reliable in quiet, are largely obscured by interfering sounds (Cooke, 2006; Cutler et al., 2008). By contrast, a different class of explanations attributes the extra difficulty of non-native listeners under adverse listening conditions to issues at higher processing levels. On such an account, energetic and informational masking by interfering sounds disrupts phonemic identification comparably for both native and non-native listeners, but the more detrimental effects are due to cumulative effects of interfering sounds on non-native listeners’ access to and integration of various types of linguistic information such as prosodic, semantic, syntactic and pragmatic cues (Bradlow and Alexander, 2007; Golestani et al., 2009; Calandruccio et al., 2010; Oliver et al., 2012).

In recent years, the explanations highlighting non-native listeners’ deficiency in multiple linguistic cues have gained support from a growing number of studies. For example, in a direct test of the two classes of explanations discussed, Cutler et al. (2004) used meaningless syllable-sized stimuli to isolate segmental information from contextual information. The results showed similar declines in phonemic identification with decreasing signal-to-noise ratios (SNRs) for both native and non-native listeners, indicating that interfering sounds do not have disproportionate effects at the segmental level if lexical- and sentence-level factors are irrelevant. These results dovetail perfectly with the findings that non-native listeners benefited less than native listeners from sentence-level contextual information for word recognition in suboptimal conditions (Bradlow and Alexander, 2007; Oliver et al., 2012).

While various types of linguistic information might contribute to the disadvantage of non-native listeners in degraded speech recognition, most of the previous studies focused on semantic context with the influences of other factors, especially prosodic cues, unexplored. As one of the most important prosodic cues, fundamental frequency (F0) has many functions in speech. In a tonal language like Chinese, F0 information is primarily used to distinguish lexical meanings (Wang, 1973; Ho and Bryant, 1997). This contrasts with non-tonal languages such as English in which variation in F0 is mainly used to mark pragmatic meanings such as emphasis, sentence modality (declarative vs. interrogative), and emotion (Wang, 1973; Repp and Lin, 1990; Cutler et al., 1997). There is ample evidence that native and non-native Chinese speakers process F0 variations differently due to the inherent differences in linguistic/paralinguistic status of pitch across the languages (Gandour et al., 2000; Xi et al., 2010; Lin and Francis, 2014). Although tones have a lexical status in Chinese, alteration of F0 patterns does not lead to unintelligibility of words in some situations. For example, synthesized Mandarin sentences with the original tone of each syllable substituted for a flat tone or a tone randomly selected from the four lexical tones could be recognized nearly perfectly by native Mandarin speakers in quiet (Wang et al., 2013; Chen et al., 2014). However, when presented in adverse listening conditions such as in a background of speech-shaped noise or against a competing talker, Mandarin speech with altered F0 patterns was substantially less intelligible than speech with natural F0 contours (Wang et al., 2013; Chen et al., 2014). Together with findings of studies on non-tonal languages, these results indicate that flattened or inverted F0 contours deteriorate speech intelligibility under adverse listening conditions regardless of whether the target language is a tonal language or not, but the effect is more detrimental for tonal languages (Laures and Bunton, 2003; Binns and Culling, 2007; Miller et al., 2010; Patel et al., 2010; Wang et al., 2013). The explanations proposed for these findings maintain that dynamic changes in F0 help with the separation of voiced/unvoiced speech segments and segmentation of words in continuous speech, and direct the listener’s attention to the content words of the utterance. Flattening or inverting F0 contours lower intelligibility of the utterance in adverse listening conditions because such manipulations alter the contrast between words and makes it more difficult to parse continuous speech into meaningful units (Laures and Bunton, 2003; Binns and Culling, 2007). This effect is more detrimental for tonal languages than non-tonal languages because in tonal languages original tones have to be recovered and mapped onto the long-term phonological representations before the lexical meaning is accessed. This process recruits specific neural and cognitive resources in which the left planum temporale and other brain areas participate (Xu et al., 2013).

It is well-known that prosodic features and lexical tones are rather difficult for L2 learners to acquire in both perception and production (Wang et al., 1999; Pennington and Ellis, 2000; Wong and Perrachione, 2007; Kang et al., 2010; Swerts and Zerbian, 2010; Huang and Jun, 2011). Therefore, the deficiency in the use of prosodic cues especially in combination of F0 information by L2 learners might have detrimental effects on non-native speech recognition, particularly under adverse listening conditions. Because previous studies on speech recognition by non-native listeners only used sentences with normal F0 contours, it is impossible to determine whether and to what extent F0 information affect speech recognition by non-native listeners.

The aim of the present study was to complement the current knowledge on speech recognition by L2 learners with respect to several points. First, given that English had been the target language in most previous studies, through examining Mandarin speech recognition by L2 learners in quiet and in the presence of an interfering single-talker speech background we aimed to extend existing research to test the generalizability of previous finding that non-native listeners benefit less from semantic context under adverse listening conditions than in quiet. Second and more importantly, we intended to explore the influence of F0 contours and the possible interaction between F0 contours and other factors, specifically semantic context and listening condition on speech recognition by L2 learners, which have rarely been examined in previous research. Finally, we were interested in obtaining evidence on whether the influences of F0 contours, semantic context and listening condition on speech recognition jointly or interactively change over time with increasing L2 proficiency of the learners. Few previous studies have been conducted on whether L2 proficiency modulates the interaction effects among these factors on L2 speech recognition.

## Materials and Methods

### Subjects

Sixty undergraduate participants from Beijing Language and Culture University were recruited. The participants were all native Japanese speakers learning Mandarin as an L2. They were thirty freshmen with elementary proficiency in Mandarin Chinese and thirty juniors with intermediate proficiency. One participant was omitted from the final analysis due to facility breakdown during data collection. The remaining 59 participants had hearing sensitivity ≤20 dB hearing level for octave frequencies between 250 and 8000 Hz bilaterally. Written informed consent was obtained from all participants. The study was approved by the Institutional Review Board (IRB) of the National Key Laboratory of Cognitive Neuroscience and Learning at Beijing Normal University. A mixed-design was adopted with F0 contours (normal vs. flat) and proficiencies in Mandarin (elementary vs. intermediate) as between-subject factors, while semantic context (sentence vs. word list) and listening condition (quiet vs. SNR = +5 dB) as within-subject factors. The participant distribution in the between-subject conditions was as follows: elementary Mandarin proficiency/normal F0, number of subjects (*n*) = 15 including 11 males and four females; elementary Mandarin proficiency/flat F0, *n* = 14 including eight males and six females; intermediate Mandarin proficiency/normal F0, *n* = 15 including 10 males and five females; intermediate Mandarin proficiency/flat F0, *n* = 15 including nine males and six females.

### Stimuli

**FIGURE 1:**
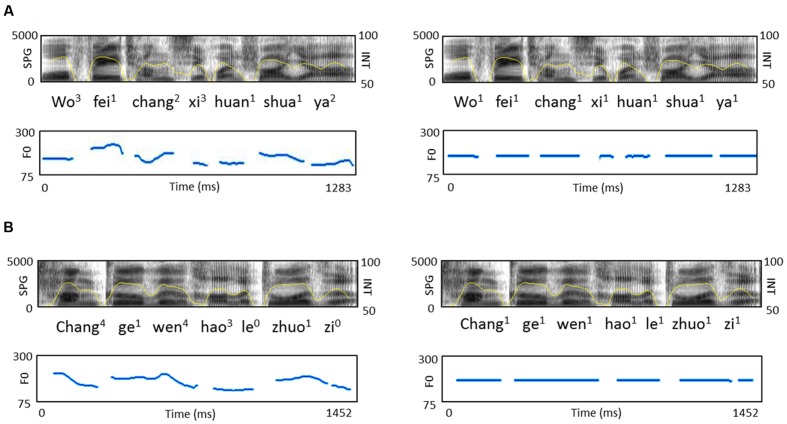
**Acoustic features of sample speech stimuli.** Broadband spectrograms (SPG: 0–5 kHz), intensity envelopes (INT: 50–100 dB), and fundamental frequency contours (F0: 0–300 Hz) are displayed for **(A)** normal sentence and its pitch-flattened counterpart; **(B)** word list sentence and its pitch-flattened counterpart.

In order to manipulate the F0 contours and semantic context effects, four types of target sentences were created, including normal sentences and word list sentences with naturally intonated or unnaturally flattened contours. Similar manipulations have been applied in our previous study (Wang et al., 2013), but in the current study we adapted the previous stimuli due to the L2 listeners’ limited proficiency in Mandarin. The normal sentences were 28 declarative Chinese sentences with each sentence comprised of 3–6 words that were familiar to the L2 learners of both levels of proficiency. Words from the entire pool of the normal sentences were pseudo-randomly selected to form the word list sentences, which were syntactically anomalous and semantically meaningless at the whole sentence level. They were matched in length (number of syllables) with the normal sentences. The normal sentences and word list sentences were read by a male native speaker of Chinese (pitch range: 90–236 Hz). Manipulation of F0 was done using Praat (Institute of Phonetic Sciences, University of Amsterdam; downloadable at www.praat.org). A flat F0 contour was created for each sentence at the sentence’s mean F0 and the resulting monotonous sentence was resynthesized using the PSOLA method (**Figure 1**).

As in our previous study of the Mandarin speech intelligibility test on adult native speakers of Mandarin Chinese (Wang et al., 2013), consonant-misplaced sentences were used as masker stimuli in order to minimize the effects of informational masking because consonant-misplaced sentences were syntactically anomalous and unintelligible at both lexical and sentential levels (Xu et al., 2013). These sentences were constructed by replacing the onset consonant of each syllable in the normal sentences with another consonant, provided that the replacement did not violate the phonotactic rules of Mandarin. The masker sentences were read by a female native Mandarin speaker (pitch range: 159–351 Hz). The choice of a male target speaker and a female masking speaker was to enable the clear instruction “listen to the male speaker” to be used throughout. Without this indexical information, participants would have to be trained on the identity of the target speaker.

Each target sentence was combined with masker noise at the SNR level of +5 dB with the target and masker sentences fixed at 75 and 70 dB sound pressure level (SPL) respectively. The masker speech was edited to be, on average, 500 ms longer than the target speech in order to ensure that no part of the speech target was unmasked.

### Procedures

We followed the same experimental procedures in our previous study (Wang et al., 2013). Specifically, listeners were tested individually in a sound-attenuated booth with ambient noise level below 15 dB(A). The stimuli were presented via loudspeakers (Edifier R18, Edifier Technology, Co. Ltd., Beijing, China). The sound level of the stimuli was calibrated to 65 dB SPL at the subject’s head. Because semantic context and listening condition were within-subject factors, each listener was presented with a total of 56 trials—14 normal sentences and 14 word list sentences in two listening conditions. Listeners were instructed that they would be listening to sentences in a quiet or interfering background, and were asked to write down the words read by the male speaker in Pinyin (a system for transcribing Mandarin with the Latin alphabet). The task was self-paced; listeners pressed a key to advance from one trial to the next. Each sentence could be heard only once. The participants were allowed to repeat the stimuli once and were instructed to guess which words they heard. Their written responses were recorded by one author of the current study (LZ). Incorrect or omitted words were annotated, and the scoring results were checked by an independent researcher blind to the experiment (who is also knowledgeable about phonology/linguistics). Practice sentences (not used in the real experiment) were provided before the experiment, which represented samples of all conditions. After the practice block, the experimenter checked the readability of the participant’s handwriting.

Only when all the phonological features, i.e., consonant, vowel and tone were correctly identified, the answer was considered correct. Speech recognition accuracy was determined by a keyword-correct count (Scott et al., 2004; Wang et al., 2013; Zhang et al., 2014). The number of keywords (content words, varied across sentences from 3 to 5) identified correctly by each listener was counted and then converted to the percentage of the total number of words and averaged across listeners. A 2 × 2 × 2 × 2 repeated measures analysis of variance (ANOVA) was carried out, with sentence context and listening condition as the within-subject factors and F0 contours and L2 proficiency as the between-subject factors.

Short-term memory was also measured by using the Chinese version of forward digit span from the WAIS III (Wechsler, 1997). In this task sets of 3–12 digits were presented to participants at the rate of 1 digit per second, and participants were asked to recall in the same order in which they heard the digits. The score was represented by the longest string that the participant correctly reported.

## Results

Scores of the Chinese version of forward digit span for the four between-subject groups are as follows: 6.8 ± 2.0 and 7.5 ± 1.5 for the two groups with elementary Mandarin proficiency; 7.2 ± 2.1 and 7.5 ± 1.9 for the two groups with intermediate Mandarin proficiency. There were no significant main effects or interaction effects across the groups [main effect of L2 proficiency: *F*(1,55) = 0.236, *p* > 0.1, η^2^ = 0.004; main effect of F0 contours: *F*(1,55) = 2.791, *p* = 0.1, η^2^ = 0.048; interaction effect between L2 proficiency and F0 contours : *F*(1,55) = 0.993, *p* > 0.1, η^2^ = 0.018]. These results showed that participants assigned to the four between-subject groups were equally matched in Mandarin short-term memory, indicating that difference in speech recognition accuracy across groups were not due to discrepancy in short-term memory.

**FIGURE 2:**
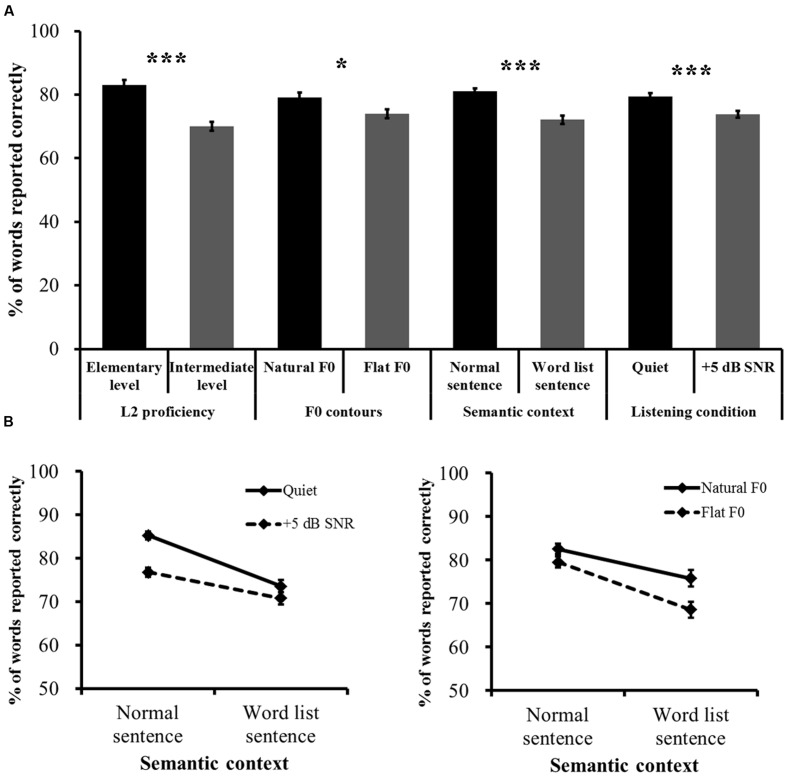
**Word-report scores sorted by the main effects and interaction effects. (A)** Main effects. **(B)** Significant two-way interaction effects. Error bars represent one standard error. ^∗^*p* < 0.05, ^∗∗∗^*p* < 0.001.

Speech recognition accuracy results showed that the four main effects were all significant [semantic context: *F*(1,55) = 86.747, *p* < 0.001, η^2^ = 0.612; listening condition: *F*(1,55) = 60.181, *p* < 0.001, η^2^ = 0.522; F0 contours: *F*(1,55) = 6.512, *p* = 0.014, η^2^ = 0.106; L2 proficiency, *F*(1,58) = 41.707, *p* < 0.001, η^2^ = 0.431], revealing that all the factors influenced Mandarin speech recognition when L2 listeners are engaged in the task. Specifically, Mandarin speech recognition accuracy improved with increasing L2 proficiency, which was compromised by the lack of sentence context and natural F0 contours and by the presence of interfering speech. Further analyses comparing all possible two- and three-way and the four-way ANOVAs revealed no significant interactions other than the following two-way interactions: the interaction effect between semantic context and F0 contours [*F*(1,55) = 4.813, *p* = 0.032, η^2^ = 0.08] revealed that recognition of normal sentences degraded to a less extent than word list sentences when the F0 patterns of the two types of sentences changed from natural contours to flat contours; the interaction effect between semantic context and listening condition [*F*(1,55) = 17.982, *p* < 0.001, η^2^ = 0.246] revealed that recognition of normal sentences degraded to a greater extent than word list sentences when listening condition changed from quiet to the interfering background (**Figure 2**). Four follow-up simple effects tests on each of the significant interactions revealed that recognition accuracy was significantly different for most contrasting pairs [*p*-values < 0.0125, Bonferroni corrected with significance threshold set at α = 0.05]. The exceptional pair was normal sentence with natural contours vs. normal sentence with flat contours [*F*(1,55) = 3.054, *p* = 0.086; see Appendix Tables A1 and A2 for the results of the simple effects tests].

## Discussion

The present study explored Mandarin speech recognition by Japanese learners of Mandarin at elementary and intermediate levels of proficiency. The overall results showed that all the factors examined in the study, i.e., semantic context, F0 contours, listening condition and L2 proficiency, affected L2 speech recognition. As the primary goal of this study was to assess the influences of semantic context and F0 contours on Mandarin speech recognition by non-native listeners in adverse listening conditions, our results revealed divergent patterns for the two factors. Specifically, there was significant modulation effect of listening condition on semantic context, indicating that L2 listeners benefited less from semantic context under adverse listening conditions than in quiet. In contrast, there was no significant modulation effect of listening condition on F0 contours. Furthermore, none of these effects were found to be modulated by L2 proficiency.

Previous work has shown that L2 learners are less accurate than native listeners in speech recognition under adverse listening conditions and this disadvantage is in large part ascribed to the deficient use of semantic context by non-native listeners (Mayo et al., 1997; Golestani et al., 2009; Oliver et al., 2012). The current results extend these findings by demonstrating that recognition of normal sentences degraded to a greater extent than word list sentences when listening condition changed from quiet to an interfering background for both groups of non-native listeners. These results are in stark contrast to those of our previous study with native Mandarin speakers (Wang et al., 2013), in which recognition of word list sentences degraded to a larger extent than normal sentences when listening condition changed from quiet to interfering backgrounds, highlighting the contribution of sentential-semantic context to speech recognition in competing speech by native listeners. The inefficient use of sentential-semantic context by non-native listeners against interfering speech might be related with their insufficient knowledge of and experience with L2, which results in a bottleneck of processing resources when listening to speech in adverse listening conditions. Furthermore, it is widely agreed that L1 processing is automatic involving a neutrally committed system to the sound patterns of the native language (Zhang et al., 2005; Zhang and Wang, 2007), but L2 processing is highly controlled and effortful (Dekeyser, 2001; Segalowitz and Hulstijn, 2005; Oliver et al., 2012), especially at elementary and intermediate levels. Thus, it is possible that during non-native speech recognition under adverse listening conditions, linguistic and cognitive processing resources are lacking for an automatic and efficient semantic processing. That is, the decrease in benefits of semantic context during L2 speech recognition under adverse listening conditions is due to capacity limitations of the non-native listeners. The result that effect of semantic context was not modulated by L2 proficiency is also consistent with the results by Mayo et al. (1997) and Shi (2010), which showed that even L2 listeners who have spoken the L2 since infancy cannot reach monolingual speakers’ level of performance on auditory sentence comprehension, especially under suboptimal listening conditions.

Although, F0 plays a crucial role in speech recognition by native listeners under adverse listening conditions (Laures and Bunton, 2003; Binns and Culling, 2007; Miller et al., 2010; Patel et al., 2010; Wang et al., 2013), how and whether it affects L2 speech recognition, especially for a tonal language, have rarely been explored. The significant main effect of F0 contours and absence of interaction effect between F0 contours and listening condition jointly revealed that natural F0 contours contributed to speech recognition by L2 listeners in both the quiet and interfering backgrounds. Furthermore, simple effect tests on the interaction between semantic context and F0 contours showed that recognition of word list sentences degraded significantly when the F0 patterns changed from natural contours to flat contours. The key difference between the two types of word list sentences is whether there are natural F0 patterns of the original tones. Taken together, these results indicate that both sentence-level (prosody) and word-level (lexical tone) F0 information could be used by L2 listeners during Mandarin speech recognition. The contribution of F0 contours to Mandarin speech recognition by L2 learners might be attributed to the development of sensitivity to Mandarin-specific F0 patterns and reflect, at least in part, the acquisition of lexical tones by non-native listeners at both elementary and intermediate levels of Mandarin proficiency. Neither the main effect of F0 contours nor the interaction effect between F0 contours and semantic context was found to be modulated by L2 proficiency. In this study, however, even the L2 listeners with elementary Mandarin proficiency had studied Mandarin for about 8 months. Whether and how sentence-level (prosody) and word-level (lexical tone) F0 information can be used by L2 listeners with lower levels of Mandarin proficiency needs to be further investigated. One interesting issue that also needs further investigation is whether native language of the participants (Japanese, a pitch-accent language which also uses variations in pitch to differentiate words) would have affected our findings, especially the use of F0 information during speech recognition in the interfering background. Future studies can be designed to compare Mandarin learners with different types of native languages, e.g., Thai (tonal language) and English (non-tonal language) to explore the generalizability of current results to Mandarin L2 speech recognition.

Interestingly, there was significant interaction between semantic context and F0 contours, i.e., recognition of normal sentences degraded to a less extent than word list sentences when F0 patterns of the two types of sentences changed from natural contours to flat contours. This result indicates that L2 listeners benefited more from semantic context when F0 information is degraded, which is in sharp contrast to the finding that they benefited less from semantic context in the interfering background than in quiet. The divergent modulation effects of listening condition and F0 contours on semantic context might be related to the differences in the distortions caused by interfering speech and flat F0 contours to the target speech signals. Specifically, interfering speech adds masking to the recognition task (Calandruccio et al., 2010) while flattening F0 contours creates distortion to the target speech itself (Miller et al., 2010; Wang et al., 2013). A recent functional magnetic resonance imaging study revealed that different neural and cognitive resources are recruited during the processing of external (e.g., background noise) and internal (e.g., accent) distortions during auditory sentence comprehension with the bilateral inferior frontal areas responsible for processing external distortions and the left temporal areas responsible for processing internal distortions (Adank et al., 2012). Future studies are needed to examine whether distinct neural substrates are involved in L2 speech recognition in the presence of interfering backgrounds and absence of natural F0 information.

In this study, single-talker babbles, specifically, consonant-misplaced sentences read by a female speaker were used as masker stimuli. One concern might arise with the potential effects of the specific masks on the current findings. Different types of masking stimuli such as single- and multi-talker babbles, white noise and speech-shaped noise have been used in previous studies on speech recognition in adverse listening conditions (Scott et al., 2004; Binns and Culling, 2007; Bradlow and Alexander, 2007; Golestani et al., 2009; Calandruccio et al., 2010; Patel et al., 2010; Oliver et al., 2012). Various interfering sounds tend to produce different masking effects, e.g., single-talker interferers usually produce less masking than multiple-talker babbles. Furthermore, in the current study, male voice was always the target and female voice was always the masker. This gender difference in the material could potentially affect our results given that male voice has lower F0 than female voice in general. More studies adopting a counterbalance design with either female or male voice served as target and different types of interfering sounds are needed in order to better understand Mandarin L2 speech recognition under suboptimal conditions.

## Conclusion

Our results support the general finding in the literature that non-native listeners benefit less from semantic context under adverse listening conditions than in quiet and, at the same time, provide initial evidence for the contribution of F0 contours and modulation effect of semantic context on F0 contours during L2 speech recognition for a tonal language irrespective of listening backgrounds. The discrepancy in the influences of semantic context and F0 contours on L2 speech recognition in the interfering background might be related to differences in processing capacities required by the two types of information in adverse listening conditions.

## Author Contributions

LZ, YL, HS, YZ, and PL designed research; LZ, YL, HW, and XL performed research; LZ, YL, and YZ analyzed data; LZ, HS, YZ, and PL wrote the paper.

## Conflict of Interest Statement

The authors declare that the research was conducted in the absence of any commercial or financial relationships that could be construed as a potential conflict of interest.
