# Supplementary material for: Effects of Semantic Context and Fundamental Frequency Contours on Mandarin Speech Recognition by Second Language Learners
Source: Front Psychol. 2016 Jun 14;7:908. doi: 10.3389/fpsyg.2016.00908 (PMC4905971; doi:10.3389/fpsyg.2016.00908)
Supplement: Supplementary file 1 [file Data_Sheet_1.DOCX]

**Appendix**

TABLE 1 Results of the four simple effects tests on the significant interaction between semantic context and listening condition.

| Simple effects | *p* | η^2^ |
| --- | --- | --- |
| NSIQ vs. NSII | 0.001 | 0.592 |
| WSIQ vs. WSII | 0.009 | 0.119 |
| NSIQ vs. WSIQ | 0.001 | 0.660 |
| NSII vs. WSII | 0.001 | 0.318 |

Abbreviations: NSIQ, normal sentence in quiet; NSII, normal sentence in the interfering background; WSIQ: word list sentence in quiet; WSII, word list sentence in the interfering background.

TABLE 2 Results of the four simple effects tests on the significant interaction between semantic context and F0 contours.

| Simple effects | *p* | η^2^ |
| --- | --- | --- |
| NSNF vs. NSFF | 0.086 | 0.053 |
| WSNF vs. WSFF | 0.008 | 0.122 |
| NSNF vs. WSNF | 0.001 | 0.319 |
| NSFF vs. WSFF | 0.001 | 0.542 |

Abbreviations: NSNF, normal sentence with natural F0 contours; NSFF, normal sentence with flat F0 contours; WSNF, word list sentence with natural F0 contours; WSFF, word list sentence with flat F0 contours.
